# Acute respiratory distress syndrome: we can’t miss regional lung perfusion!

**DOI:** 10.1186/s12871-015-0014-z

**Published:** 2015-03-18

**Authors:** Paolo Pelosi, Marcelo Gama de Abreu

**Affiliations:** 1 Department of Surgical Sciences and Integrated Diagnostics, IRCCS San Martino IST, University of Genoa, Genoa, Italy; 2 Department of Anesthesiology and Intensive Care Medicine, Pulmonary Engineering Group, University Hospital Carl Gustav Carus; Technische Universität Dresden, Dresden, Germany

**Keywords:** Acute respiratory distress syndrome, Pulmonary blood flow, Ventilator induced lung injury, Gas exchange, Lung imaging

## Abstract

In adult respiratory distress syndrome (ARDS), life-threatening hypoxemia may occur, dictating the need for differentiated ventilator strategies. Pronounced consolidation and/or atelectasis have been well documented in ARDS, but the contribution of regional perfusion to oxygenation has been poorly addressed. Evidence has accumulated that, in ARDS, regional perfusion is extremely variable and may affect oxygenation, independently from the amount of atelectatic-consolidated lung regions. Thus, the response in oxygenation to different ventilatory settings, both during controlled and assisted mechanical ventilation, should be interpreted with caution. In fact, gas exchange may be not determined solely by changes in aeration, but also redistribution of perfusion. Furthermore, regional perfusion can play an important role in worsening of lung injury due to increased transmural pressures. In addition, distribution of perfusion in lungs might affect the delivery of drugs through the pulmonary circulation, including antibiotics. In recent years, several techniques have been developed to determine pulmonary blood flow with increasing level of spatial resolution, allowing a better understanding of normal physiology and various pathophysiological conditions, but most of them are restricted to experimental or clinical research. Lung ultrasound and novel algorithms for electrical impedance tomography represent new promising techniques that could enable physicians to assess the distribution of pulmonary blood flow at the bedside. In ARDS, we cannot afford missing regional lung perfusion!

Please see related article: http://dx.doi.org/10.1186/s12871-015-0013-0.

## Background

The acute respiratory distress syndrome (ARDS) is an acute life-threatening condition, triggered by different clinical disorders, and characterized by severe hypoxemia refractory to increased inspired oxygen fraction, and high mortality [1]. Hypoxemia is closely related to the consolidation or collapse of lung regions, which increase the intrapulmonary shunt. Accordingly, most strategies aimed at improving oxygenation in ARDS target at the improvement of aeration and ventilation.

Different imaging techniques such as computed tomography [2], electrical impedance tomography [3] and lung ultrasonography [4] have been used to investigate the distribution of aeration and/or ventilation in ARDS. However, arterial oxygenation results from the coupling between alveolar ventilation and perfusion. Therefore, a favorable distribution of regional perfusion could prevent deterioration of oxygenation, even in presence of non-aerated lung tissue.

## Main text

Richter et al. [5] investigated the distribution of regional pulmonary blood flow in a model of acute lung injury induced by hydrochloric acid in rats. They found that 2 hours after injury, the pulmonary blood flow shifted away from injured regions, explaining at least in part a progressive improvement in oxygenation. These data confirm the importance of regional pulmonary blood flow for the pathophysiology of ARDS, while providing valuable insight into the clinical management of patients suffering from this syndrome.

The role of pulmonary perfusion in ARDS is important for different reasons. First, perfusion potentially interacts with and modulates ventilator induced lung injury. Second, it is a major determinant of pulmonary gas-exchange. Third, it reflects pulmonary vascular resistance, heart-lung interactions, venous return to the heart, and even systemic hemodynamics [6]. Fourth, experimental studies showed that a higher transmural pressure, which results from the pressure gradient between the alveoli-interstitium and intracapillary pressure, promotes higher epithelium and endothelium injury, as well as lung edema [7,8].

Different mechanisms can explain shifts in regional perfusion, including obstruction or compression of pulmonary capillaries due to swelling of endothelial cells, and pulmonary hypoxic vasoconstriction. In ARDS patients, redistribution of pulmonary blood flow contributes only slightly to maintenance of oxygenation [9], suggesting that hypoxic vasoconstriction is blunted, which differs from the data presented by Richter et al. [5]. Possible explanations for this discrepancy are differences in the etiology of ARDS, species, as well as type of ventilation, i.e. assisted vs. spontaneous.

During pressure support ventilation, improvements in oxygenation were associated with a redistribution of perfusion towards well-aerated, non-dependent lung regions, without evident alveolar recruitment [10,11]. In contrast, biphasic positive pressure ventilation with additional mandatory breaths [12,13], as well as variable controlled ventilation [14], promoted redistribution of perfusion towards dependent lung regions, and alveolar recruitment. Differences in the pattern of redistribution of perfusion might be explained by: 1) regional pleural pressures during inspiration; 2) mean airway pressure. In fact, time cycling compared to flow cycling promotes higher mean airway pressure yielding better lung stabilization. The redistribution of regional pulmonary blood flow can explain at least in part possible beneficial effects of assisted ventilation on ventilator-induced lung injury [15-17].

Ideally, assessment of lung perfusion should be performed in real-time. Unfortunately, such methods are not available at bedside yet. For research, different techniques have been proposed, including single photon emission computed tomography, positron emission tomography [18], and multi-detector row computed tomography [19]. For clinical practice, a new electrical impedance tomography-based method that quantitatively estimates regional lung perfusion based on first-pass kinetics of a bolus of hypertonic saline contrast has been reported [20-22]. In addition, color Doppler ultrasound might also give information about regional perfusion in non-aerated lung regions [23,24].

### Discussion

The better understanding of the role of regional perfusion could have important implications for daily clinical practice. First, the response in oxygenation to different ventilatory settings should be interpreted always with caution, since they might be not determined by lung recruitment or derecruitment, but redistribution of perfusion. Second, an excessive pulmonary blood flow, either global or regional, can contribute to ventilator-induced lung injury; thus, optimal fluid balance and adequate monitoring should be mandatory. Third, low tidal volume should be used to prevent or minimize ventilator-induced lung injury, and positive end-expiratory pressure preferably titrated on respiratory mechanics changes than oxygenation. A more detailed and comprehensive approach, including bedside lung imaging, hemodynamics, gas-exchange and mechanics should be preferred, at least in most severe cases. Fourth, due to the unpredictable distribution of blood flow, it is likely that drugs delivered to the lungs, like intravenous antibiotics, will not reach in adequate amount the affected regions, possibly compromising their effectiveness, specially in assisted ventilation. We believe that the evaluation of regional perfusion at the bedside should be implemented more often to follow the course of the disease and clinical treatment.

## Conclusions

The experimental study by Richter et al. [5] provides further evidence that regional perfusion affects ventilator-induced lung injury, and importantly modulates gas-exchange in ARDS. In recent years, several techniques have been developed to determine pulmonary blood flow with increasing level of spatial resolution, allowing a better understanding of normal physiology and various pathophysiological conditions, but most of them are restricted to experimental or clinical research. Lung ultrasound and novel algorithms for electrical impedance tomography represent new promising techniques that could enable physicians to assess the distribution of pulmonary blood flow at the bedside. In ARDS, we cannot afford missing regional lung perfusion!

## Abbreviations

ARDS: Acute respiratory distress syndrome
